# Mapping loci and genes controlling heading and maturity time in common wheat under long-day conditions and assessing their effects on yield-related traits

**DOI:** 10.18699/vjgb-25-84

**Published:** 2025-10

**Authors:** A.A. Kiseleva, A.I. Stasyuk, I.N. Leonova, E.A. Salina

**Affiliations:** Institute of Cytology and Genetics of the Siberian Branch of the Russian Academy of Sciences, Novosibirsk, Russia; Institute of Cytology and Genetics of the Siberian Branch of the Russian Academy of Sciences, Novosibirsk, Russia; Institute of Cytology and Genetics of the Siberian Branch of the Russian Academy of Sciences, Novosibirsk, Russia; Institute of Cytology and Genetics of the Siberian Branch of the Russian Academy of Sciences, Novosibirsk, Russia

**Keywords:** common wheat, heading time, maturity time, yield traits, QTL mapping, Ppd-D1, Vrn-B3, пшеница, время колошения, время созревания, урожайность, QTL картирование, Ppd-D1, Vrn-B3

## Abstract

The duration of the vegetation period significantly impacts yield formation and is one of the important characteristics of spring common wheat (Triticum aestivum L.) varieties. The primary developmental phases influencing the vegetation period include the time from seedling emergence to heading and from heading to maturity. To identify genes and loci associated with these traits under long-day conditions typical of Western Siberia and to assess their impact on yield components, we conducted QTL mapping followed by an evaluation of yield-related traits in lines carrying different alleles of key heading time genes. For mapping, we used an F2 population derived from a cross between the varieties Obskaya 2 and Tulun 15, which contrast in their heading and maturity times. QTL analysis identified a novel locus on the long arm of chromosome 7B associated with maturity time, as well as two loci on chromosome 2D and the short arm of chromosome 7B associated with heading time. Gene analysis within these loci revealed candidate genes for the “seedling-maturity” trait, with expression patterns corresponding to the known maturity time regulator NAM-1. The localization of loci for the “seedling-to-heading” trait suggested their correspondence to the well-known genes Ppd-D1 and Vrn-B3. Analysis of progeny carrying the Ppd-D1a and Vrn-B3a allele combination demonstrated that Ppd-D1a had a stronger effect on heading time than Vrn-B3a, and their combined presence resulted in the earliest heading – on average, five days earlier than in lines with the Ppd-D1b and vrn-B3 alleles. Evaluation of yield-related traits (number and weight of grains per main spike and per plant, and 1,000-grain weight) indicated that Ppd-D1 was significantly associated with all traits, with the Ppd-D1a allele generally exerting a negative effect on yield. In contrast, Vrn-B3 had a comparatively smaller effect on yield traits than Ppd-D1.

## Abstract (ru)

Продолжительность вегетационного периода оказывает значительное влияние на формирование урожая и является одной из важных характеристик сортов яровой мягкой пшеницы (Triticum aestivum L.). К основным межфазным периодам, влияющим на продолжительность вегетации, относятся время от всходов до колошения и от колошения до созревания. Для выявления генов и локусов, ассоциированных с этими признаками в условиях длинного дня, характерного для Западной Сибири, и оценки их влияния на структуру урожая мы провели картирование QTL с последующей оценкой признаков урожайности у линий, несущих различные аллели генов, определяющих скорость развития. В качестве картирующей популяции мы использовали растения F2, полученные от скрещивания контрастных по скорости развития сортов Обская 2 и Тулун 15. QTL анализ выявил новый локус на длинном плече хромосомы 7B, ассоциированный со временем созревания, и два локуса на хромосоме 2D и коротком плече хромосомы 7B, ассоциированных со временем колошения. Анализ генов, входящих в состав локусов, позволил выявить гены-кандидаты для признака «всходы–созревание», паттерны экспрессии которых соответствовали генам известного регулятора скорости созревания NAM-1. Локализация локусов для признака «всходы–колошение» позволила предположить, что они соответствуют известным генам Ppd-D1 и Vrn-B3. Анализ потомства линий с сочетанием аллелей Ppd-D1a и Vrn- B3a показал, что Ppd-D1a оказывает больший эффект на время колошения, чем Vrn-B3a, а сочетание этих двух аллелей приводит к наиболее раннему колошению, в среднем на пять дней раньше линий с аллелями Ppd-D1b и vrn-B3. Оценка признаков структуры урожая (количество и масса зерен с главного колоса и с растения, масса 1000 зерен) показала, что ген Ppd-D1 ассоциирован со всеми признаками на высоком уровне значимости, при этом в большинстве случаев аллель Ppd-D1a негативно влиял на урожайность. Ген Vrn-B3 влиял на признаки урожайности в меньшей степени по сравнению с Ppd-D1.

## Introduction

Wheat yield and its adaptability to diverse environmental conditions are largely determined by the duration of key developmental stages. The most critical stages in wheat development are the transition from vegetative to reproductive growth – namely, heading time – and maturity time. In variety evaluations, the prediction of heading time is most often based on the allelic composition of key regulatory genes, including Vrn-A1, Vrn-B1, Vrn-D1, Ppd-D1, and, less frequently, Vrn-B3 (TaFT-1), as these genes exert the most substantial influence on this trait (Zhang Y. et al., 2010; Kiss et al., 2014; Chen S. et al., 2018; Mizuno et al., 2022; Palomino, Cabrera, 2023). The primary genes associated with maturity time are those belonging to the NAM-1 family (Hagenblad et al., 2012; Alhabbar et al., 2018b).

Most Ppd-1 alleles conferring photoperiod insensitivity are characterized by structural changes in the promoter region, such as deletions or insertions, which affect various regulatory sequences (Beales et al., 2007; Wilhelm et al., 2009; Nishida et al., 2013). Besides, dominant alleles known for Ppd-B1 are characterized by an increased number of copies (Díaz et al., 2012). Among all Ppd-1 genes, the dominant Ppd-D1a allele is currently the most widely utilized in global wheat breeding programs (Seki et al., 2011). According to Z. Guo et al., the Ppd-D1a allele has been identified in 33 % of common wheat cultivars in South America, 45.5 % of those cultivated in Southern Europe, and 8 % of varieties in Northern and Western Europe (Guo et al., 2010). The highest frequency of this allele has been reported in Asia, where it is present in 57.4 % of wheat cultivars grown in China. Among Japanese cultivars, 84 % carry this dominant allele (Seki et al., 2011). In contrast, the Ppd-D1a allele remains relatively rare among cultivars developed through Russian breeding programs, despite its potential not only to accelerate heading but also to positively influence other agronomic traits (Likhenko et al., 2014; Lysenko et al., 2014). In most studies, the effect of this allele has been examined under short-day conditions, where it shortens the time to heading by 20 to 30 days. However, there is limited evidence suggesting that the Ppd-D1a allele can also accelerate heading by 3 to 5 days even under long-day conditions (Worland et al., 1998; Kiseleva et al., 2014).

Another important gene is Vrn-B3, which serves as a central regulator of heading time. The Vrn-B3a allele promotes early flowering, with its expression enhanced by the insertion of a 5,300 bp retroelement into the promoter region (Yan et al., 2006). This allele is very rarely found in cultivated wheat varieties (Zhang X.K. et al., 2008; Iqbal et al., 2011; Chen F. et al., 2013; Lysenko et al., 2014). Additionally, four other alleles of this gene – designated as b, c, d, and e – have been identified, although they exhibit much weaker effects on The wild-type allele of the NAM-B1 gene is associated with earlier maturity; however, it is rarely found in modern cultivars due to its negative impact on yield (Lundström et al., 2017). Alleles of its homoeolog, NAM-A1 – specifically NAM-A1a and NAM-A1b – have also been identified and are similarly associated with earlier maturity (Alhabbar et al., 2018a).

Environmental conditions significantly influence the developmental rate of common wheat. Climatic conditions vary considerably across different regions where wheat is cultivated in Russia. Despite the clear importance of investigating the regulation of heading and maturity times under long-day conditions – typical of most regions in Russia – the genetic mechanisms underlying these traits under such photoperiods remain poorly understood. For example, several studies have demonstrated the influence of Vrn-1, Vrn-B3, and NAM-A1 on maturity time (Zaitseva, Lemesh, 2015; Alhabbar et al., 2018a; Whittal et al., 2018). However, in our previous research, no association was found between the allelic state of these genes and maturity time in spring wheat cultivars under the conditions of Western Siberia. Instead, novel loci associated with this trait were identified on chromosomes 2A, 3B, 4A, 5B, 7A, and 7B (Kiseleva et al., 2023).

Thus, the genetic control of wheat developmental rates is highly dependent on growing conditions, with different genes influencing the trait in distinct climatic zones. In Western Siberia, there is a particular need for early-maturing, highyielding common wheat cultivars, as most varieties currently registered for this region are mid-season types. Cultivating spring wheat with a range of maturity times allows for a more flexible harvest schedule, which is crucial for minimizing yield losses due to over-ripening (Belan et al., 2021). This highlights the need for further analysis of known loci and identification of new loci and genes controlling the duration of key growth stages, as well as breeding of wheat cultivars and lines with heading and maturity times adapted to specific environmental conditions

The objective of our study was to identify loci and their associated genes related to the duration of major developmental phases in spring common wheat and to assess their effects on yield under the environmental conditions of Western Siberia

## Materials and methods

Plant material. The mapping population was developed from a cross between two spring common wheat cultivars, Obskaya 2 and Tulun 15. Obskaya 2 belongs to the group of mid-season cultivars and is characterized by high yield potential and baking quality comparable to that of premium wheat. Tulun 15 is an early-maturing cultivar with high grain quality, although it has lower yield performance compared to Obskaya 2. Hybridization of the parental cultivars, subsequent self-pollination of the F1 hybrids, and cultivation of the F2 generation were carried out under the greenhouse conditions of the Federal Research Center Institute of Cytology and Genetics, Siberian Branch of the Russian Academy of Sciences (ICG SB RAS). The subsequent F3 and F4 generations were also obtained through self-pollination and grown under field conditions (Fig. S1)1.


Supplementary Materials are available in the online version of the paper: https://vavilovj-icg.ru/download/pict-2025-29/appx26.xlsx


Phenotypic analysis. The F3 and F4 generation plants derived from the cross between Obskaya 2 and Tulun 15 were sown in 2018 and 2019 at the experimental field of the Siberian Research Institute of Plant Production and Breeding (Krasnoobsk, Novosibirsk Region; 54.914070°N, 82.975379°E)

Heading was defined as the stage when half of the wheat spike had emerged from the sheath, and heading time was recorded as the duration (in days) from seedling emergence to heading. Maturity was determined based on the hardening of the grain and the yellowing and drying of the spikes and stems. The grain filling period was calculated as the difference between maturity time and heading time. The soil at the experimental field was leached chernozem. Field trials were established on plots 0.5 meters wide, with 20 seeds sown per row. Each sample was sown in two rows with a 20 cm row spacing. Mature plants were harvested in bundles, dried, and subsequently used for yield component analysis. The number of grains per main spike, the grain weight per main spike, the total number and weight of grains per plant, and the 1000-grain weight were evaluated. Structural analysis was performed on 20 plants per sample.

Weather conditions in the Novosibirsk Region during the growing seasons deviated from the long-term averages. In May 2018, the mean monthly temperature was 7 °С, compared to the long-term average of 12.5 °С, while rainfall reached 82 mm – 2.5 times higher than the norm. In June, July, and August 2018, temperatures were close to long-term averages. Rainfall in June and July did not differ significantly from the norm; however, August was characterized by warm and dry conditions, with only 35 mm of rainfall compared to the average of 53 mm. In 2019, the temperature regime throughout the growing season was consistent with long-term averages. Moisture availability in May 2019 was comparable to the long-term norm. However, June and August experienced a moisture deficit, with only 26 mm and 22 mm of precipitation, respectively, compared to the averages of 59 mm and 53 mm. In contrast, July 2019 was warm and humid, with 98 mm of rainfall compared to the average of 69 mm.

DNA extraction and PCR. Genomic DNA was extracted from wheat leaf tissue using a modified protocol based on the method published by J. Plaschke et al. (Plaschke et al., 1995). Allele-specific primers previously reported in the literature were used to identify the alleles of the Vrn-1, Vrn-B3, and Ppd-D1 genes (Yan et al., 2004, 2006; Fu et al., 2005; Beales et al., 2007; Shcherban et al., 2012). PCR amplification was performed using a T100 Thermal Cycler (Bio-Rad, USA) and BioMaster HS-Taq PCR-Color reagents (Biolabmix, Russia) following the protocols described in the corresponding publicationsGenotyping, genetic map construction, and QTL mapping. High-throughput SNP genotyping was performed on DNA from the F2 mapping population of common wheat (84 lines) derived from the cross between Obskaya 2 and Tulun 15, using the Illumina Infinium 20K Wheat chip (TraitGenetics GmbH, Germany). A total of 17,267 markers were analyzed.

Genetic map construction was performed using the MultiPoint UltraDense software (Mester et al., 2003). Markers with more than 25 errors or with a segregation distortion (χ2) greater than 42 were removed. The minimum size for a group of co-segregating markers (linked markers mapped to the same position) was set at 2. Clustering was carried out with a recombination fraction threshold of 1.5. Marker ordering within clusters was conducted using the GES (guided evolutionary strategy) algorithm with jackknife resampling. To generate stable maps, monotony control was applied, involving the removal of outlier markers followed by sequential elimination of destabilizing markers

Using the constructed genetic maps and phenotypic data, QTL mapping was conducted to identify loci controlling heading time, maturity time, and grain filling period in the population derived from the cross between Obskaya 2 and Tulun 15. QTL mapping was performed using the MultiQTL software based on the CIM (composite interval mapping) algorithm

Gene prioritization from identified loci. For the functional characterization of candidate genes, sequences were annotated using the IWGSC RefSeq v1.0 reference genome. Gene expression patterns were assessed based on transcriptome sequencing data from the common wheat cultivar Azhurnaya, obtained from various plant tissues during development from germination to full maturity (Ramírez-González et al., 2018). As reference transcripts, sequences for NAM-A1 (TraesCS6A02G108300.1/. 2) and NAM-D1 (TraesCS6D02G096300.1) were used, since NAM-B1 in the Chinese Spring (CS) cultivar is represented by a non-functional allele and does not have an annotated ID in the RefSeq genome annotation

Statistical analysis. Descriptive statistics, ANOVA, Tukey test, and Pearson correlation coefficients (r) were calculated using R software. All plots were generated with the R package ggplot2 (Wickham, 2016).

## Results

**Evaluation of developmental phase duration in the mapping population**

**Fig. 1.**
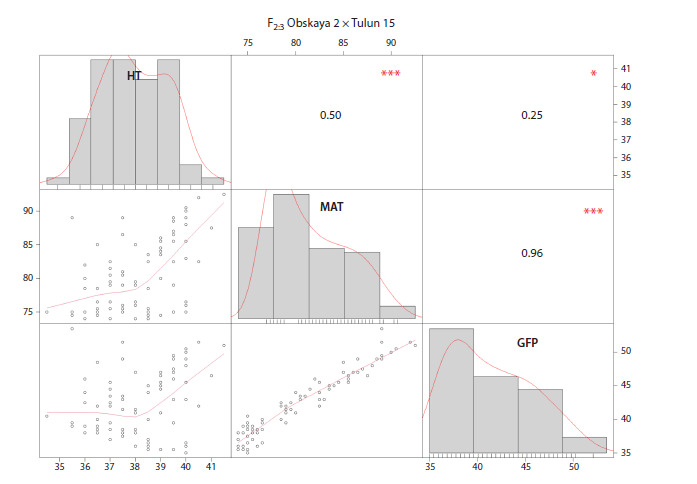
Pearson correlation coefficients between traits related to developmental rate. HT – heading time; MAT – maturity time; GFP – grain filling period. Significant differences are indicated by asterisks: * p < 0.05; *** p <0.001.

Heading time in the population ranged from 34 to 41 days. Descriptive statistics for the three traits are presented in Table S1. The distribution of heading time was approximately normal (Fig. 1), while the distributions of maturity time and grain filling period were skewed toward lower values. A very strong correlation was observed between maturity time and grain filling period, with an r2 of 0.96.

**Genetic maps and QTL mapping**

For the construction of genetic maps for the Obskaya 2 × Tulun 15 population, 3,323 polymorphic markers were selected. Of these, 2,629 markers were mapped, including 534 skeleton markers. A total of 25 linkage groups were developed, with chromosomes 3B, 3D, and 5D represented by multiple groups. Summary data are presented in Table S2, and graphical representations of the maps are shown in Figure S2.

**Fig. 2.**
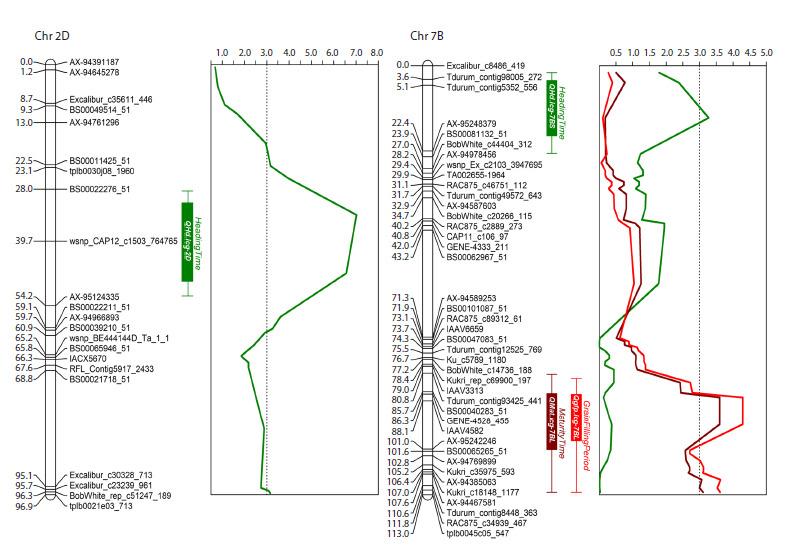
Genetic maps of chromosomes 2D and 7B (showing only skeleton markers) with loci associated with heading time (green), maturity time (maroon), and grain filling period (red) indicated.

Using the CIM model, significant loci associated with the duration of developmental phases in common wheat were mapped to chromosomes 2D and 7B (Fig. 2). The locus on chromosome 2D was associated exclusively with heading time and accounted for 37 % of the phenotypic variation (PEV) for this trait. A locus on the short arm of chromosome 7B was also associated with heading time, explaining 20 % of the variation. Additionally, two loci on the long arm of chromosome 7B were associated with maturity time and grain filling period; these loci overlapped. The PEV values were 11.5 % for maturity time and 18 % for grain filling period.

The locus on chromosome 2D associated with heading time was located in the interval between markers BS00022276_51 (position 29454345 on RefSeq v1.0) and wsnp_CAP12_ c1503_764765 (position 35683599 on RefSeq v1.0). Thus, the most likely candidate gene for this QTL is Ppd-D1 (position 33952048–33956269), the physical location of which on the reference map precisely corresponds to the interval between the identified markers.

The locus on chromosome 7B associated with heading time was located in the interval between markers Tdurum_ contig5352_556 (position 5061935 on RefSeq v1.0) and AX-95248379 (position 12717101 on RefSeq v1.0). Thus, the most likely candidate gene for this QTL is Vrn-B3 (position 9702354–9704354), the physical location of which on the reference map falls within the interval defined by these markers

For the loci associated with maturity time and grain filling period on the long arm of chromosome 7B, no known genes were identified. A search of the WheatQTLdb database also did not reveal any loci with similar positions.

**Candidate genes within the maturity time and grain filling period locus**

The QMat.icg-7BL locus was localized to the interval between 712618516 and 721195460 bp on RefSeq v1.0 and contains 141 genes (Table S3). Analysis of gene expression patterns in various tissues during plant development identified several candidate genes (Table S4).

A total of eight genes were identified that are predominantly expressed in the flag leaf, the fifth leaf after heading, or in the grain: TraesCS7B02G455300, TraesCS7B02G459500, TraesCS7B02G459600, TraesCS7B02G460500, TraesCS7B02G460300, TraesCS7B02G454000, TraesCS7B02G461300, and TraesCS7B02G461400.

**Selection of plants with different alleles of Ppd-D1 and Vrn-B3**

The Ppd-D1 and Vrn-B3 genes were identified through QTL analysis as the primary candidates controlling heading time in the studied population. Therefore, PCR was conducted to determine their allelic composition. Genotyping revealed that the early-maturing cultivar Tulun 15 carries the Ppd-D1a and Vrn-B3a alleles, whereas Obskaya 2 carries the Ppd-D1b and vrn-B3 alleles. Both cultivars shared the same allelic composition for the Vrn-1 genes: Vrn-A1a, Vrn-B1c, and vrn-D1.

Subsequently, the F2 population plants were genotyped. As a result, 34 plants in which Ppd-D1 and Vrn-B3 were in a homozygous state were selected. Based on their allelic composition, the selected plants were divided into four groups (Fig. S1). The F3 and F4 progeny of these plants were sown in the field to determine the heading times for each group.

**Evaluation of heading time in the F3 and F4 populations**

**Fig. 3.**
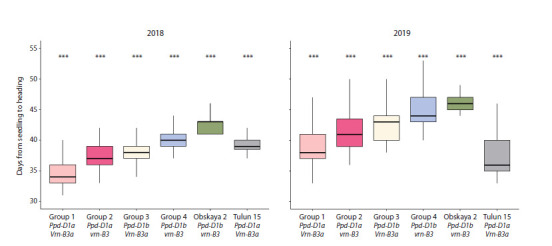
Comparison of the duration from seedling to heading in F3 and F4 plants derived from the cross between Obskaya 2 and Tulun 15. *** Significant differences between hybrids and parental cultivars at p <0.001.

Evaluation of the period from seedling emergence to heading in 2018 showed that plants from Group 1, carrying the dominant alleles Ppd-D1a and Vrn-B3a, headed the earliest – on average at 34.5 days (Fig. 3). Plants carrying the Ppd-D1a allele and the recessive vrn-B3 allele (Group 2) headed 2.8 days later than Group 1, at 37.3 days. Plants from Group 3 (Ppd- D1b and Vrn-B3a) headed 38 days after emergence. The latest heading was observed in Group 4 plants (40.3 days), in which both genes were in the recessive state. Although the parental cultivar Obskaya 2 had the same allelic composition as Group 4, it headed even later, at 42.9 days. The Tulun 15 cultivar, despite carrying the same allelic combination as Group 1 (Ppd-D1a and Vrn-B3a), headed 3 days later than the Group 1 plants.

Evaluation of the duration from seedling emergence to heading in 2019 showed that the shortest period (37.1 days) was observed in the parental cultivar Tulun 15 (Fig. 3). Plants from Group 1, which had the same allelic composition for the Vrn and Ppd genes, headed 39 days after emergence, which was 5.8 days earlier than the plants from Group 4 (homozygous for the recessive vrn-B3 and Ppd-D1b alleles). In Group 2, carrying the dominant Ppd-D1a allele and the recessive vrn-B3 allele, the heading period was 2.5 days longer than in Group 1, totaling 41.5 days. Even later heading (42.8 days) was recorded in Group 3, where plants carried the recessive Ppd-D1b allele and the dominant Vrn-B3a allele. Among the hybrid population, plants in Group 4, with the recessive Ppd-D1b and vrn-B3 alleles, headed the latest – 44.8 days after emergence. The parental cultivar Obskaya 2 exhibited the longest period from emergence to heading – 46.8 days.

ANOVA confirmed that the alleles of Ppd-D1 and Vrn-B3 significantly influenced heading time in both years of the study (Table S5). The presence of the Ppd-D1a allele accelerated heading by 3.5 days in 2018 and by 4.4 days in 2019. The Vrn-B3a allele accelerated heading by 2.3 days in 2018 and by 2.5 days in 2019. Moreover, the combination of these two alleles resulted in heading 5.5 days earlier in 2018 and 6.5 days earlier in 2019 compared to the combination of the recessive alleles of these genes

**Effect of the Ppd-D1 and Vrn-B3 alleles on yield components**

In both years, several yield parameters were evaluated, including the number of grains per main spike, grain weight per main spike, total number and weight of grains per plant, and 1000-grain weight (Table S6). According to ANOVA results, group classification based on the combination of Ppd-D1 and Vrn-B3 alleles had a significant effect on all traits, while the year of cultivation significantly affected all traits except for the number of grains per plant and grain weight per plant (Table S7).

The number of grains per main spike in the Obskaya 2 cultivar was the highest in 2018 – averaging 39.25 grains – and was significantly greater ( p < 0.001) than in all four studied groups. The lowest number of grains per spike was observed in plants from Group 1 (25.65 grains), which differed significantly from Group 3 (29.08 grains) and Group 4 (28.56 grains). In Group 2 and in the Tulun 15 cultivar, the number of grains per spike was 26.83 and 31.60, respectively; however, no significant differences were detected between these and other groups. In 2019, significant differences were observed only between Group 1 (38.68 grains) and Group 3 (43.67 grains).

The grain weight per main spike in the Obskaya 2 cultivar was significantly higher than in all other plants in the experiment in both years: 1.99 g in 2018 and 2.21 g in 2019. Plants of the Tulun 15 cultivar had the lowest grain weight per spike in both 2018 (0.94 g) and 2019 (1.38 g). However, in 2018, there were no significant differences between Tulun 15 and the four groups, whereas in 2019, significant differences were observed between Tulun 15 and Group 3 (1.76 g). Comparing the groups, in 2018, Groups 1 (1.04 g) and 2 (1.02 g) had significantly lower grain weight per spike compared to Groups 3 (1.25 g) and 4 (1.20 g). In 2019, the grain weight per spike in Groups 1 (1.51 g) and 2 (1.40 g) was also significantly lower than in Group 3 (1.76 g).

The number of grains per plant in 2018 was the lowest in Group 1 (45.54 grains) and differed significantly compared to Group 2 (63.18 grains), Group 3 (66.52 grains), Group 4 (59.91 grains), and the Obskaya 2 cultivar (72.30 grains). No significant differences were observed between Group 1 and the Tulun 15 cultivar (54.60 grains). In 2019, there were no significant differences among all the studied plants for this trait

The grain weight per plant in 2018 was highest in Obskaya 2 (3.56 g) and differed significantly from all other groups. Significant differences were also observed between Group 1 plants (1.78 g) and those in Group 3 (2.71 g) and Group 4 (2.44 g). In 2019, the Obskaya 2 cultivar again showed a significantly higher grain weight per plant (3.53 g) compared to all other plants in the experiment. The grain weight per plant in Groups 1, 2, 3, 4, and in Tulun 15 was 2.03, 2.13, 2.39, 2.28, and 2.34 g, respectively; however, no significant differences among these groups were detected in 2019.

The highest 1000-grain weight was observed in the Obskaya 2 cultivar in both years – 48.95 g in 2018 and 45.94 g in 2019 – and was significantly greater than in all other plants in the experiment. The lowest 1000-grain weight was recorded in the Tulun 15 cultivar: 29.58 g in 2018 and 34.15 g in 2019. In 2018, significant differences were observed between Tulun 15 and plants from Groups 1 (38.86 g), 2 (36.51 g), and 4 (38.62 g), while in 2019, significant differences were found between Tulun 15 and Group 3 (39.28 g). In 2018, no significant differences were found between Group 1 (38.86 g) and Groups 2 (36.51 g), 3 (40.96 g), and 4 (38.62 g), or between Groups 3 and 4. In 2019, no significant differences were detected between Group 1 (37.70 g) and Groups 3 (39.28 g) and 4 (37.15 g). However, the 1000-grain weight of the Group 2 plants (32.93 g) was significantly lower than that of Groups 1, 3, and 4.

All these observations were supported by factorial analysis, where the allele combinations of Ppd-D1 and Vrn-B3 were used as factors (Table S7). ANOVA revealed that the allelic state of Ppd-D1 was significantly associated with most of the evaluated traits in both years of the study, with a high level of significance (except for the number of grains per plant in 2019). In contrast, the allelic state of Vrn-B3 demonstrated a lower level of significance for all traits compared to Ppd-D1. In 2019, Vrn-B3 showed a significant association only with the 1000-grain weight. Overall, it can be concluded that the lines from Groups 3 and 4 possess a higher yield potential compared to other groups, although still lower than that of the original cultivar Obskaya 2.

## Discussion

**A new locus associated with maturity time**

The correlation between maturity time and grain filling period was very high (0.96), indicating that in this population and under long-day conditions, grain filling period contributes the most to overall maturity time rather than heading time, despite the range for heading time being about seven days – a considerable variation. Previous studies have also reported that maturity time does not always depend on heading time and grain filling period and may be influenced by independent mechanisms (May, Van Sanford, 1992; Kajimura et al., 2011). However, genes specifically associated with maturity time in common wheat, apart from the NAM-1 gene family, are largely unknown (Hagenblad et al., 2012).

In this study, we identified the QMat.icg-7BL locus on the long arm of chromosome 7B, associated with maturity time, localized within the interval 712618516–721195460 bp (RefSeq v1.0). Although a considerable number of studies have focused on identifying markers and loci associated with maturity time, describing loci on most wheat chromosomes except 3A and 6A, they have resulted in only a few associations for this trait being reported for chromosome 7B (Kulwal et al., 2003; McCartney et al., 2005; Huang et al., 2006; Kamran et al., 2013; Yu et al., 2015; Perez-Lara et al., 2016; Zou et al., 2017). We hypothesized that this locus might coincide with a previously described maturity time locus identified through GWAS in a population of domestic spring wheat cultivars (Kiseleva et al., 2023). However, the QTL mapped in the present study was located closer to the telomere and did not overlap with the previously identified locus. Similarly, comparison with another previously reported locus on chromosome 7B associated with maturity time (Kulwal et al., 2003) also revealed no overlap. Thus, we can conclude that we have identified a novel locus associated with maturity time.

Within the boundaries of this locus, 141 genes were identified. Based on the analysis of expression patterns in various tissues during plant development, several candidate genes associated with maturity time were identified. The TraesCS7B02G455300 gene exhibited an expression pattern most similar to that of NAM-A1 and NAM-D1, with a peak in the flag leaf at the full maturity stage. This gene encodes 12-oxophytodienoate reductase 1, a key enzyme involved in jasmonic acid biosynthesis. Previously, it was described as one of the candidate genes involved in the regulation of stem density (Taria et al., 2025). The TraesCS7B02G459500, TraesCS7B02G459600, TraesCS7B02G460500, and TraesCS7B02G460300 genes also exhibited expression in the flag leaf after the heading stage and showed similar expression patterns in the fifth leaf. TraesCS7B02G454000 showed increased expression during maturation in the fifth leaf and had detectable expression in the first leaf only at the tillering stage. TraesCS7B02G461300 and TraesCS7B02G461400 are annotated as Pseudo-Response Regulators, belonging to the same gene family as Ppd-1, one of the main genes regulating heading time. These genes exhibited relatively low expression levels but were specific to the grain at the milk and dough stages of development

**The Ppd-D1a and Vrn-B3a alleles significantly influence heading time under long-day conditions**

With the same allelic combination of Vrn-A1a, Vrn-B1c, and vrn-D1, the presence of the dominant Ppd-D1a and Vrn- B3a alleles results in the earliest heading under long-day conditions. When the dominant Ppd-D1a allele is combined with the recessive vrn-B3 allele, heading time is delayed by 2.5–3 days. In plants carrying the recessive Ppd-D1b allele and the dominant Vrn-B3a allele, the emergence-to-heading period is further extended by an additional 1–1.3 days.

The obtained results indicate that the Ppd-D1a allele exerts a stronger influence on the rate of transition to the generative phase of wheat development than the Vrn-B3a allele. This finding is consistent with the QTL analysis results, which showed that the locus on chromosome 2D explains a larger percentage of the variation in this trait.

Plants in which both genes are in the recessive form (Group 4) transition to heading significantly later than plants from the other groups. The Obskaya 2 cultivar, which also carries the recessive Ppd-D1b and vrn-B3 alleles, heads even later – by an additional 2 to 2.5 days. Although the QTL analysis did not reveal other significant loci associated with heading time, this could be due to the presence of minor loci that were not detected with sufficient significance in the analysis, and that may have been inherited by Group 4 plants from the early-maturing cultivar Tulun 15. In addition to the genes studied here, other known genes such as TaELF3, PhyC, PhyB, WPCL, and numerous QTLs distributed across all chromosomes have been shown to influence the transition rate to the generative phase (Chen A. et al., 2014; Milec et al., 2014; Mizuno et al., 2016; Pearce et al., 2016; Wang et al., 2016; Zikhali et al., 2016).

When comparing heading time assessments across the two years (Fig. 3), it can be observed that in the second year of the study, heading occurred 4 to 5 days later in all hybrid groups and in the Obskaya 2 cultivar. The exception was the Tulun 15 cultivar, in which the duration period from emergence to heading remained unchanged at 37 days. This increase in heading time is likely due to differences in weather conditions between 2018 and 2019. According to data from gismeteo.ru, the average air temperature in June 2018 was 21.3 °C, whereas in June 2019 it was 18.5 °C – 2.8 °C lower. The stability of heading time in the Tulun 15 cultivar may indicate its high level of environmental plasticity.

We observed that the effects of combining the Ppd-D1a and Vrn-B3a alleles are additive (Table S5). According to the established model of floral meristem initiation leading to heading (Li C. et al., 2024), Ppd-D1 acts as a primary inducer of the Vrn-B3 gene. It is possible that Ppd-D1a does not directly influence Vrn-B3a because Vrn-B3a expression is already enhanced due to promoter region modifications (insertion events). Thus, it can be hypothesized that in the presence of the Vrn-B3a allele, Ppd-D1a affects heading time through the Vrn-3 homeologs located on chromosomes 7A and 7D. Although few studies have specifically investigated the involvement of these genes in heading time regulation, (Bonnin et al., 2008) demonstrated that nucleotide polymorphisms in Vrn-A3 and Vrn-D3 were associated with variations in heading time.

**Effect of early heading alleles on other agronomically important traits**

The effects of Ppd-1 genes on various agriculturally significant traits have been demonstrated in several previous studies. For example, (Boden et al., 2015) showed that these genes play a crucial role in inflorescence architecture and the development of paired spikelets in wheat. A more complex influence of Ppd-1 on spike traits, including spike length, number of spikelets, and anther length, has also been reported (Okada et al., 2019), as well as effects on tiller number and spikelet number (Li W.L. et al., 2002). Several studies have further noted the influence of Ppd-1 on the number of grains per main spike and 1000-grain weight (Wu et al., 2021). Our results showed that the Ppd-D1a allele had a significant negative effect on traits such as the number and weight of grains per spike and per plant, as well as on 1000-grain weight.

There is limited information available on the effects of Vrn- 3 genes on these traits. According to our data, the effect of the Vrn-B3a allele on the traits studied was less pronounced compared to Ppd-D1a, and its expression was more strongly influenced by growing conditions.

In most cases, when the differences were significant ( p-value < 0.001), the dominant alleles of the studied genes were associated with lower trait values (fewer and lighter grains). Moreover, the trait values for the parental cultivar Tulun 15 were even lower, suggesting the involvement of additional genetic mechanisms regulating these traits independently of the duration of the vegetative phase.

**Selection of lines from different maturity groups with high productivity traits**

Among the F4 generation plants, a search was conducted for highly productive lines across all four groups differing in heading time. Selection was based on traits such as grain weight per plant, which reflects yield potential, and 1000-grain weight, which reflects grain size. Additionally, visual assessment of the plants in the field was taken into account. It is well established that wheat yield has a strong correlation with the length of the vegetative period. In our experiment, variation was observed within each group for both grain weight per plant and 1000-grain weight. Although Group 1, characterized by the earliest heading, generally exhibited lower productivity traits, some early-heading lines from this group demonstrated grain weight per plant and 1000-grain weight values comparable to those of Group 4, which had the latest heading time, and substantially exceeded the early-maturing parental cultivar Tulun 15. Lines with good trait values were also identified in Group 3. In Group 2, several lines showed competitive grain weight per plant; however, the 1000-grain weight was the lowest among all groups. Based on this analysis, 19 lines from all four groups were selected as promising candidates for further breeding efforts (Table S8).

## Conclusion

The results obtained allow us to conclude that the Ppd-D1a and Vrn-B3a alleles have a significant impact on the heading time of spring common wheat under long-day conditions. Ppd-D1a accelerates heading more strongly but also exerts a more pronounced negative effect on traits related to productivity. It is hypothesized that in the presence of the Vrn-B3a allele, Ppd-D1a may influence heading time through its homeologs Vrn-A3 and Vrn-D3. The practical significance of this study lies in the development of new promising breeding lines of spring wheat with heading times optimized for many regions of Russia and other parts of the world characterized by long photoperiods.

## Conflict of interest

The authors declare no conflict of interest.
